# Vertebrate Adaptive Immunity—Comparative Insights from a Teleost Model

**DOI:** 10.3389/fimmu.2017.01379

**Published:** 2017-10-26

**Authors:** Harry W. Dickerson, Robert Craig Findly

**Affiliations:** ^1^Department of Infectious Diseases, College of Veterinary Medicine, University of Georgia, Athens, GA, United States

**Keywords:** *Ichthyophthirius multifiliis*, channel catfish, teleost, adaptive immunity, immune memory, T cell repertoire

## Abstract

The channel catfish (*Ictalurus punctatus*) and the ciliated protozoan parasite *Ichthyophthirius multifiliis* are used to study pathogen-specific protective immunity. In this review, we briefly describe this host–parasite system and discuss the comparative insights it provides on the adaptive immune response of vertebrates. We include studies related to cutaneous mucosal immunity, B cell memory responses, and analyses of αβ T cell receptor (TCR) repertoires. This host–parasite model has played an important role in elucidating host protective responses to parasite invasion and for comparative studies of vertebrate immunity. Recent findings from bioinformatics analyses of TCR β repertoires suggest that channel catfish preferentially expand specific clonotypes that are stably integrated in the genome. This finding could have broad implications related to diversity in lymphocyte receptors of early vertebrates.

## Introduction

The comparative immunology of early vertebrates has developed from basic descriptive studies to investigations using mechanistic experimentation, molecular genetic analyses, and bioinformatics (1). Studies with teleosts (bony fishes) are useful in providing insights into the evolution of the adaptive immune responses of higher vertebrates, including humans (2). The channel catfish (*Ictalurus punctatus*) and the ciliated protozoan parasite *Ichthyophthirius multifiliis* have been used as an experimental system to study pathogen-specific adaptive immunity (3). Laboratory-induced *I. multifiliis* infections in catfish are particularly suited for comparative studies of mucosal immunity because the parasite is restricted to surface epithelia of the gills and skin, which in teleosts are protected by a mucosal layer. Investigations with this model have elucidated cutaneous adaptive immune responses to infection and other basic elements of immunity, including memory and repertoire generation, which are discussed in this review.

## Channel Catfish—An Established Fish Model

Although primarily recognized as a food fish raised for human consumption, the channel catfish has served as a comparative immunological research model for more than 30 years, providing a foundation of basic information on the molecular and cellular basis of both innate and adaptive immunity in teleosts (4–26). The channel catfish serves as an excellent comparative biological model for studies of early ectothermic vertebrate immunity. The recent publication by Liu et al. (25) presents the genomic sequence of the channel catfish and illustrates its early phylogenetic position among teleosts. Similar to other fishes including salmonids and zebrafish (*Danio rerio*), which is one of the best known model fish species in the biomedical research field, the thymus and head kidney of channel catfish are the primary lymphoid organs in which T and B cells differentiate, respectively. The spleen serves as a secondary peripheral lymphoid organ. The head kidney is the site of hematopoiesis and serves as both a primary and secondary lymphoid organ. Although putative primordial germinal centers are found in head kidney and spleen, there are no lymph nodes, and organized lymphoid tissues have not been identified in mucosal tissues including those of the skin and gut epithelia (27). Thymus-derived, cell-dependent, immune functions including hapten-carrier, graft rejection, and delayed hypersensitivity were all demonstrated in early studies on immune responses in channel catfish, as well as B and T cell proliferation *in vitro* in response to mitogens (6). As with other fish models, there are limited data regarding T cell surface marker genes (28, 23).

The channel catfish IgH gene locus is organized in a translocon-type arrangement with a single functional C gene that codes for membrane and secreted forms of IgM (7, 11). The diversity within channel catfish VH genes may be greater than that found in humans (20). The predominant serum IgM is a tetrameric molecule, homologous to the mammalian IgM isotype, comprised of 70,000 Da heavy chains (IgH) and 22,000–25,000 Da light (IgL) chains in equimolar quantities (10). The tetramer has a molecular mass of approximately 750,000 Da. When analyzed by SDS-PAGE under denaturing conditions, however, it shows various combinations of covalently linked heavy and light chains to form eight discernable Ab subpopulations (29). The significance of this variable covalent coupling is unknown, but may relate to affinity and to diverse functions in different tissues (e.g., skin mucosal secretions) (30, 31).

The Ig surface receptor and soluble Ab homologous to the mammalian IgD isotype were first discovered in channel catfish and their subsequent characterization in other teleost fishes suggests that IgD is an evolutionarily ancient Ig with a potentially important, but as yet unknown, function (8). Studies of IgD function in humans suggest an ancient role for IgD at the interface between immunity and inflammation (32). An IgT/Z isotype found in rainbow trout (*Oncorhynchus mykiss*) and zebrafish is postulated to function in teleost fishes as the equivalent of mammalian IgA (33). Genes for IgT/Z do not occur in all fishes, however. Channel catfish does not have these genes and only expresses IgD and IgM. In channel catfish IgM provides Ab protection in mucosal tissues, including skin and gills (12, 34, 35).

The isotypes IgA, IgE, and IgG are not present in any fish species and class switching does not occur, even though teleosts express activation-induced cytidine deaminase (AID), an enzyme essential for this function. Zebrafish and catfish AID have the potential to catalyze class switch recombination, however, indicating that this capability preceded the evolution of IgA, IgE, and IgG (36, 37). AID-catalyzed somatic hypermutation has been demonstrated in fish, including channel catfish (26, 38).

Although the complex interactions between T and B cells during and following infections in teleosts have not been studied in comparable detail to that of mammals, many of the basic mechanisms appear similar. CD40 and CD154 homologs have been identified in zebrafish. CD40 is localized to B cells and *in vivo* experiments demonstrated that blocking its interaction with CD154 decreased IgM synthesis. The functional CD154-CD40 costimulatory pathway in teleosts appears similar to that in humans, and T cell help is involved in controlling antibody production by B cells (39, 40).

## Adaptive Immunity and *I. multifiliis—*A Protozoan Pathogen of Fishes

In the current paradigm of acquired immunity, re-exposure to protective antigens initiates anamnestic memory responses in secondary lymphoid tissues that lead to expansion and further differentiation of antigen-specific effector lymphocytes (41). In mammals including humans, the concept of memory cells being confined only to lymphoid tissues has been revised following the discovery that subsets of memory lymphocytes also reside in non-lymphoid tissues (42, 43). This is of particular relevance because most pathogens infect through surface epithelial tissues where they confront the hosts’ first lines of defense. A subset of memory T cells appears to reside permanently in extralymphoid tissues at potential sites of colonization and infection (43). If present in sufficient numbers at the time of infection, these non-circulating T cells can control pathogen colonization and proliferation. Circulating memory T cells are also recruited to sites of colonization as a result of inflammation. More severe infections, however, which expand beyond the sites of initial invasion, lead to activation of memory T cell subsets in secondary lymphoid tissues. These memory cells rapidly proliferate and differentiate into effector cells that migrate to sites of infection throughout the body (43). In addition to memory T cells, memory B cells and antibody-secreting plasma cells also reside at potential sites of infection, as well as in secondary lymphoid tissues, including MALT and spleen (44–47).

**Figure 1:**
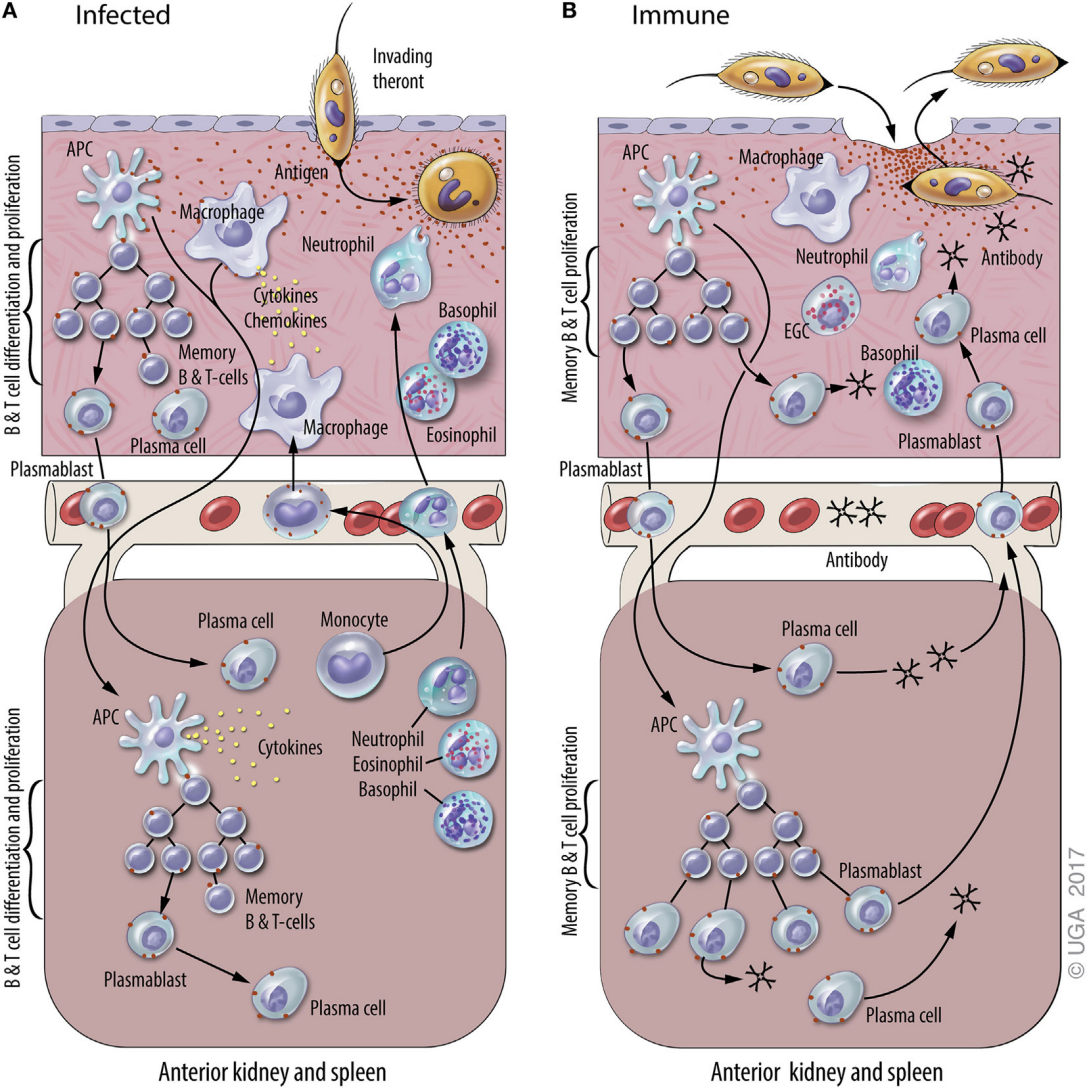
A general model of adaptive immunity against *Ichthyophthirius multifiliis* (Ich). The natural physiological state of the skin has immune components that monitor and respond to infections, including resident macrophages, putative dendritic cell subsets, and resident T and B lymphocytes. Elements of mucosal and systemic immunity against Ich (some of which are hypothetical and others based on experimental data) are presented in panels **(A)** and (**B)** of this figure. This model is based on studies of Ich infection in channel catfish and various other fish species, and the figure is derived from a previous review of Ich immunity (50). **(A)** Infection of naive fish. Parasite invasion in the skin (upper section) elicits local and systemic inflammatory responses that include the recruitment of neutrophils, basophils, and eosinophils, and the production of cytokines and chemokines. The uptake of Ich Ags by macrophages and dendritic cells (APC) initiates the adaptive immune response. Ags are processed and presented by APC at inductive sites in the skin (hypothetical) and/or the anterior kidney and spleen (lower section), where plasmablasts, plasma cells, and memory B and T cells are generated. Components of the adaptive immune response exist as early as 7–10 days after initial infection, and last up to three years after the infection is resolved. **(B)** Infection of immune fish. Parasite entry into the epithelium of the skin elicits a cellular response that includes basophils, neutrophils, and eosinophilic granulocytic cells (EGCs), which are analogous to mammalian mast cells. Ab in the skin bind to invading theronts and elicit their pre-mature exit. Ich-specific Ab in the blood and skin are secreted by plasma cells in the central lymphoid organs and the skin, respectively. We hypothesize that plasmablasts generated in central lymphoid organs and the skin traffic between both sites through the blood. Blood vessels are depicted between the upper and lower sections of each panel.

*Ichthyophthirius multifiliis*, commonly known “white spot” by home aquarists, is an obligate, ciliated protozoan parasite that infects the skin and gills of virtually all species of freshwater fishes. It occurs worldwide and is among the most devastating pathogens of both wild and domestic fish (48, 49). The life cycle lasts 7–10 days at 22°C and includes a tomont stage (200–800 µM) that replicates off the fish, the infective, actively swimming pelagic theront stage (40 µM), and an obligate trophont stage (50–800 µM) that feeds and grows within the host’s epithelia (3, 49). *I. multifiliis* infection stimulates an antibody-mediated immune response, as expected for a large, single-cell, motile, extracellular parasite. Immunity develops within 3 weeks and lasts over 3 years (12, 50, 51). *I. multifiliis* exposure elicits cutaneous mucosal immunity in channel catfish resulting from the synthesis of IgM Abs by antibody-secreting cell (ASC) located in the skin (3, 12, 18, 35, 50–52) (Figure 1). These studies on cutaneous immune responses of channel catfish to *I. multifiliis* infections were subsequently followed by studies in trout (53). The response is dominated by IgM Abs found in both skin and serum that target a class of highly abundant 40–60-kDa surface membrane proteins, referred to as immobilization antigens (i-antigens). We have shown that immunization of channel catfish with purified i-antigens is sufficient to induce protective immunity (54–56). A model for the mechanism of antibody-mediated protection against *I. multifiliis* has been proposed in which cross-linking of immobilizing Ab to i-antigens elicits exit from the host (3, 52). *I. multifiliis*, unlike African trypanosomes, which switch the expressed variant surface glycoproteins to evade the host immune responses, do not switch the expressed i-antigens that elicit protective immune responses (57). This is an example in which long-lasting immunity to a protozoan parasite is conferred by immunization with a single class of proteins. The highly effective protective immune response elicited by the parasite has served as a stimulus for basic research to elucidate the mechanisms of adaptive immunity in teleosts (3, 50, 58).

Memory T and B cells are the basis of anamnestic responses in vertebrates including fishes (59, 60). In teleosts, however, there is little information on where memory B cells and T cells reside and how stable these populations are over time. B cell responses to infection have indicated that ASC are critical to development of protective immunity against subsequent I. multifiliis challenge (12, 50, 61). Studies suggest that long-term protective immunity is provided by IgM^+^ memory B cells rather than long-lived resident, non-dividing plasma cells (12). In contrast, in humans Abs against many vaccine Ags are synthesized by plasma cells for most of, if not their entire life (62). It has not been determined whether memory B cells reside in skin and differentiate *in situ* into i-antigen-specific ASC when fish are re-exposed to *I. multifiliis*, or if they reside in other lymphoid organs, and migrate to the skin following re-exposure (12, 18, 63) (Figure 1). Because Ab reagents that define memory B or T cells are not currently available for fish, these lymphocyte sub populations cannot be separated by FACS or *in situ* staining as in humans and mice.

## αβ T Cell Receptor (TCR) Repertoires

The genes coding for the αβ TCR heterodimer in teleosts are organized in a classical translocon arrangement comprised of families of V and J genes with heterogeneous sequences, but only one or a few D and C genes (17, 19, 64–67). As in mammals, rearrangement of V, D, and J genes is dependent on *rag1* expression, and the diversity afforded by recombination of these genes is augmented by random non-templated nucleotide deletions and additions at TCRβ V–D and D–J gene junctions, or TCRα V–J gene junctions (68). The sequences at these junctions code for the hypervariable aa sequences that comprise the complementarity determining region 3 (CDR3) loops of the αβ TCR and determine its Ag recognition and binding affinity (69). In channel catfish, the TCRβ CDR3 spans 27–60 base pairs, or 9–20 aa, similar in size to the human TCRβ CDR3 (17, 19, 70–72).

The diversity of aa sequences in the anticipatory CDR3 repertoire of the population of circulating T cells allows recognition and binding to the vast array of foreign peptide Ags presented by the MHC (69). The changes that occur in expressed TCRβ CDR3 repertoires in an individual after infection with a pathogen, the variation in different tissues, and the differences among individuals responding to the same infection, are not well characterized in human patients. Because the fins of fish can regenerate (73), we used the *I. multifiliis* infection model to characterize TCRβ CDR3 repertoires in biopsies of caudal fin taken before infection and three weeks after infection. Skin, spleen, head kidney and PBL samples were collected at seven and twenty-one weeks after infection. High-throughput sequencing of cDNAs synthesized from RNA isolated from these samples was used to determine the expressed diversity of Vβ2 and Vβ5 TCRβ CDR3 repertoires, as αβ T cells expressing Vβ2 and Vβ5 genes undergo clonal expansion after infection (70).

### Public and Private Clonotypes in the Immune Repertoire

The changes in TCRβ repertoires are illustrated by results for Vβ2. We generated 1.73 × 10^6^ copies of filtered CDR3 DNA sequences, representing 1.02 × 10^5^ unique DNA sequences and 7.4 × 10^4^ aa clonotypes. A clonotype designates a unique CDR3 aa sequence. Public clonotypes are defined as those present in all individuals sampled (74). A total of 5.5 × 10^3^ clonotypes were public, which is higher than theoretically predicted for humans (69, 71, 72, 75). The diversity of the TCRβ CDR3 repertoire was found in rare sequences. DNA sequences found only once in the entire data set represented 40% of unique DNA sequences and those present in two to nine copies another 30%. These rare sequences presumably correspond to αβ T cells present in low abundance. Only a small fraction of unique DNA sequences were highly abundant. Those present in 10^2^–10^3^ copies represented only 2% of DNA sequences and those present in >10^3^ copies only 1%. This small set of DNA sequences, however, comprised the bulk of all copies with those present in >10^2^ copies contributing 63% of total copies.

### Non-Random Selection of DNA Sequences Coding for Dominant Clonotypes

**Figure 2:**
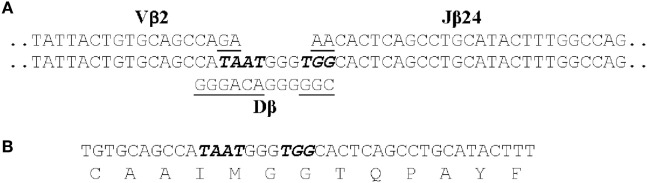
The CDR3 DNA and amino acid sequence of clonotype CAAIMGGTQPAYF. (**A)** The genomic sequences of the Vβ2, Dβ, and Jβ24 genes that contribute to the CDR3 sequence are shown. Those nucleotides that were presumably deleted from the Vβ2, Dβ, and Jβ24 genes are underlined and the seven non-templated nucleotides added are italicized. (**B)** The CDR3 nucleotide and aa sequence of the transcribed TCRβ gene for CAAIMGGTQPAYF. This same nucleotide sequence was found in cDNAs generated from four fish.

We identified 12 public clonotypes that were among the most abundant in skin or spleen of all four fish following *I. multifiliis* infection. As these clonotypes were present in low relative abundance or absent in pre-infection fin samples, we inferred that these corresponded to clonotypes expressed by clonally expanded αβ T cells responding to infection. These clonotypes could dominate the repertoire in a tissue after immunization. For instance, clonotype CAAIMGGTQPAYF accounted for 7.9% of all copies of DNA sequences in the spleen of one fish, and 5.8% in skin of a second. Because of codon degeneracy we expected that an array of different DNA sequences would code for each clonotype and that these arrays would differ among fish, as seen for TCRβ CDR3 repertoires in PBL from human donors (75). Unexpectedly, we found that each of these 12 clonotypes was predominately coded by an identical CDR3 DNA sequence in combination with the same J gene in different fish. The DNA sequence of one of these public clonotypes is shown in Figure 2. This dominant DNA sequence comprised 97% of the sequences coding for the clonotype after infection.

Following infection alternative DNA sequences coding for each clonotype were also identified. These 11–40 alternative sequences, however, represented only ~3% of total copies of DNA sequences coding for a clonotype. The alternative sequences demonstrate that αβ T cells, expressing a Vβ2 CDR3 coding for a clonotype responsive to *I. multifiliis* infection, could be generated by standard somatic recombination of Vβ2, Dβ, and Jβ genes accompanied by deletion and addition of nucleotides at V–D and D–J junctions. These αβ T cells did not persist in the populations, however, as copies of most alternative DNA sequences were not present at later time points after infection. Only those αβ T cells expressing the dominant DNA sequence apparently underwent clonal expansion. This suggests that the selection of αβ T cells expressing the dominant DNA sequence was not random (70).

## Future Work

Future studies using high-throughput sequencing will be focused on defining the CDR3 repertoire for additional Vβ genes following infection and immunization with purified i-antigens. In addition, the molecular mechanisms underlying the selective use of an identical DNA sequence by different fish to code for the same clonotype need to be defined. The exquisite diversity in the mammalian CDR3 repertoire is provided by the randomly generated DNA sequences coding for the CDR3, and there is little overlap among individuals at the DNA sequence level (75, 76). The finding that different fish preferentially expressed the same CDR3 DNA sequence for a clonotype suggests the possibility that these rearranged TCRβ sequences were stably integrated into the *I. punctatus* genome and that T cells expressing these integrated clonotypes are preferentially expanded. An integrated IgH VDJ gene sequence is present in the *I. punctatus* IgH locus, although it is not known if it is transcribed (7). Future research will focus on understanding how these identical sequences are transcribed in different individuals. Such studies will further define similarities and differences in adaptive immune responses among phylogenetically diverse vertebrate groups such as teleosts and mammals and provide further comparative insights into the evolution of vertebrate immune repertoires.

## Author Contributions

HD and RF contributed equally to the writing of the manuscript.

## Conflict of Interest Statement

The authors declare that the research was conducted in the absence of any commercial or financial relationships that could be construed as a potential conflict of interest.

## Abbreviations

Ig, immunoglobulin; aa, amino acid; Ab, antibody; Ag, antigen; ASCs, antibody-secreting cells; TCR, T cell receptor; CDR3, complementarity determining region 3; i-antigens, immobilization antigens; AID, activation-induced cytidine deaminase; MALT, mucosa-associated lymphoid tissue.
